# Highly Sensitive Quantitative Real-Time PCR for the Detection of *Plasmodium* Liver-Stage Parasite Burden following Low-Dose Sporozoite Challenge

**DOI:** 10.1371/journal.pone.0077811

**Published:** 2013-10-02

**Authors:** Sophie Schussek, Penny L. Groves, Simon H. Apte, Denise L. Doolan

**Affiliations:** 1 Infectious Diseases Programme, Queensland Institute of Medical Research, Herston, Queensland, Australia; 2 School of Medicine, University of Queensland, St Lucia, Queensland, Australia; Institut de Recherche pour le Développement, France

## Abstract

The pre-erythrocytic stages of *Plasmodium* *spp.* are increasingly recognised as ideal targets for prophylactic vaccines and drug treatments. Intense research efforts in the last decade have been focused on *in vitro* culture and *in vivo* detection and quantification of liver stage parasites to assess the effects of candidate vaccines or drugs. Typically, the onset of blood stage parasitaemia is used as a surrogate endpoint to estimate the efficacy of vaccines and drugs targeting pre-erythrocytic parasite stages in animal models. However, this provides no information on the parasite burden in the liver after vaccination or treatment and therefore does not detect partial efficacy of any vaccine or drug candidates. Herein, we describe a quantitative RT-PCR method adapted to detect and quantitate *Plasmodium yoelii* liver stages in mice with increased sensitivity even after challenge with as few as 50 cryopreserved sporozoites (corresponding to approximately 5-10 freshly isolated sporozoites). We have validated our quantitative RT-PCR assay according to the MIQE (Minimum Information for Publication of Quantitative Real-Time PCR Experiments) guidelines and established high reproducibility and accuracy. Our assay provides a rapid and reproducible assessment of liver stage parasite burden in rodent malaria models, thereby facilitating the evaluation of the efficacy of anti-malarial drugs or prophylactic vaccines with high precision and efficacy.

## Introduction

Malaria is a vector-borne disease, caused by the apicomplexan parasite *Plasmodium* *spp.* and transmitted by *Anopheles* mosquitoes. Upon taking a blood meal, an infected female mosquito injects *Plasmodium* *spp.* sporozoites into the dermis, which then migrate to the liver and go through a pre-erythrocytic developmental stage within hepatocytes before initiating the symptomatic erythrocytic stage of the infection [1,2]. The pre-erythrocytic stage represents a bottleneck in the parasite development, is clinically silent and therefore regarded as an ideal point of intervention for prophylactic treatment and vaccination strategies [3]. Inhibition of parasite growth in hepatocytes can result in reduction or complete ablation of erythrocytic stages, thus attenuating or eliminating the symptoms and the pathology of the disease as well as preventing further disease transmission. Indeed, infection with attenuated sporozoites, whose development is halted in the liver, induces sterile protective immunity [4,5].

The low percentage of host hepatocytes that are infected after inoculation with *Plasmodium* *spp.* sporozoites and the limited number of available tools pose major obstacles for the evaluation of liver-stage development and the quantitative analysis of pre-erythrocytic anti-parasite effects induced by experimental drug treatments or vaccines *in vivo* [3]. The conventional approach to evaluate the effect of an intervention on liver-stage development *in vivo*, involves assessment of the development of blood-stage parasitaemia, via the presence or absence of parasites in the blood after sporozoite infection and/or the prepatent period to onset of parasitaemia. The complete absence of blood-stage parasitaemia indicates sterile protection. However, such assessment fails to evaluate any effects directed only at the liver stage or discriminate between the liver and blood stage for interventions potentially directed at either or both parasite stages. More direct methods used to study development of the parasite in the liver *in vivo* include histopathologic examination of liver sections [6,7], flow cytometry of fluorescent protein-tagged parasites [8], intra-vital imaging of GFP or luciferase expressing transgene parasites [1,3], quantitative PCR of ribosomal DNA [9] and real-time quantitative reverse-transcription PCR (qRT-PCR) [10,11].

qRT-PCR eliminates the requirement for transgenic parasites used for fluorescence or bioluminescence methods and has demonstrated high sensitivity for the detection of other pathogens, where standard (usually microscopic) methods for quantification are problematic or not sensitive enough to detect low parasite burdens [12]. We and others have previously described qRT-PCR methods which can detect liver stage parasite burden following challenge with relatively high doses of sporozoites or bites of infected mosquitoes. However, more sensitive qRT-PCR methods are required to measure parasite load after challenge with low parasite numbers that are comparable to those of natural infection [13]. To address this, we adapted the qRT-PCR method described previously by Witney et al. [11] to develop a highly sensitive quantitative RT-PCR assay, which we validated according to the MIQE (Minimum Information for Publication of Quantitative Real-Time PCR Experiments) guidelines [14].

## Materials and Methods

### 1. Mice and parasites

Specific pathogen-free BALB/c mice (Animal Resource Centre, Perth, Australia) were used at 6-10 weeks of age. The parasite strain used in all experiments was *Plasmodium yoelii* 17X NL derived from cryopreserved *s*porozoites provided by Dr. Stephen Hoffman (Sanaria Inc., Rockville, MD, USA). Mice were infected with 50-5000 cryopreserved infectious sporozoites or 1000 heat-inactivated sporozoites in 200µl 1x PBS/ 2% naive mouse serum injected intravenous (i.v.) into the tail vein. Cryopreserved sporozoites were inactivated by incubation at 72°C for 15min and then at 95°C for 15min. For fluorescent microscopy of live or heat-inactivated sporozoites, parasites were suspended in µ-slide 18-well slides (Ibidi, Munich, Germany) with 0.1% SYTOX Blue (Molecular Probes, Mulgrave, Australia) in 1xPBS; Invitrogen, Life Technologies Australia Pty Ltd., Mulgrave, VIC). Images were captured using the DeltaVision Core Microscope System, with Coolsnap HQ camera and 100x/1.40 oil objective (Applied Precision, Issaquah, USA). Live sporozoites were motile and excluded SYTOX Blue; heat-inactivated sporozoites were all non-motile, crescent-shaped and their nuclei stained with SYTOX Blue. Mice were euthanized at 40-42h post sporozoite challenge. All studies were approved by The QIMR Animal Ethics Committee and were conducted in accordance with the Australian Code of Practice for the Care and Use of Animals for Scientific Purposes (2004).

### 2. RNA extraction

**Figure 1:**
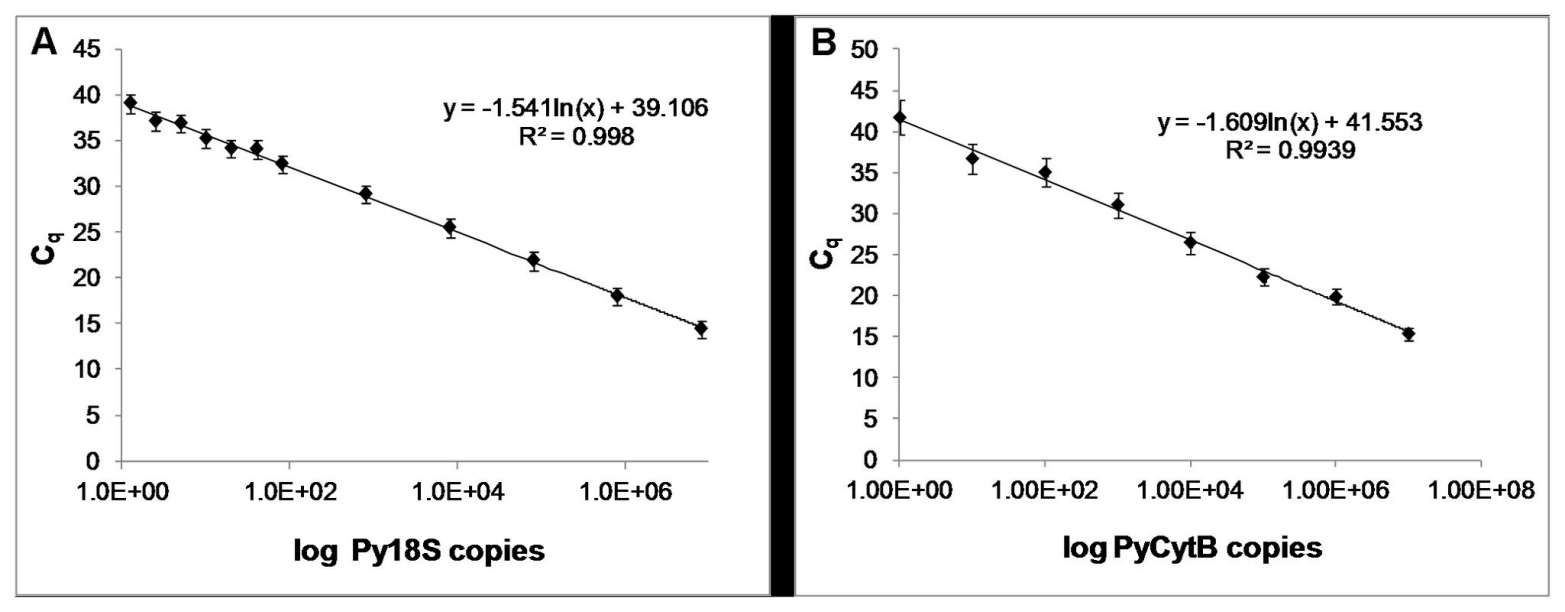
Standard Curves. qRT-PCR analysis of a dilution series ranging from 10^7^ to 1 copies of Py18S (A) and PyCytB (B) control plasmid. Data are presented as mean values for three independent runs with two technical replicates each. Error bars represent mean standard deviation between technical replicates of each run.

Whole livers were harvested 40-42h after sporozoite challenge and homogenised in 5ml RNeasy lysis buffer (RLT, Qiagen Pty Ltd., Chadstone Centre, VIC) with 1% β-2-mercaptoethanol (Sigma-Aldrich Co. LLC., St. Louis, USA). RNA was extracted from a 200µl aliquot of the whole liver homogenate (one twenty-fifth of the whole liver) using the Qiagen RNeasy Mini kit (Qiagen Pty Ltd.) according to manufacturer’s instructions. All equipment used during RNA extraction was cleaned with RNase away wipes (Thermo Scientific, VWR, Arlington Heights, USA) and exposed to UV-light for at least 2h prior to use. The A260/A280 ratio of purified RNA was typically between 1.8 and 2.4 (generally accepted ratios of A260/A280 for good quality RNA are >1.8) and the yield between 80µg and 120µg (as measured on PowerWave HT Microplate Spectrophotometer, BioTek, VT, USA). RNA samples and aliquots of the liver homogenates were stored at -80°C. RNA integrity was assessed by gel electrophoresis to determine the intensity of the large and the small subunit of ribosomal RNA using Quantity One 1-D Analysis Software (Bio-Rad Laboratories Pty. Ltd., NSW) (Figure 1A).

### 3. cDNA synthesis

cDNA was synthesised from 2.5µg RNA using SuperScript® VILO^TM^ cDNA synthesis kit (Life Technologies) according to manufacturer’s guidelines. Briefly, cDNA synthesis reactions were assembled in 20µl on ice and then incubated for 10 mins at room temperature, followed by 2h at 42°C. The reaction was terminated by heating the sample to 85°C for 5 mins. cDNA samples were stored at -20°C.

### 4. Construction of control plasmids and standard curves

Two different target sequences were evaluated: 18S rRNA, the small subunit of ribosomal RNA which is widely used in qRT-PCR assays [15,16], and cytochrome b (CytB) mitochondrial DNA (mtDNA), which has been suggested to provide increased sensitivity through higher abundance [17]. *P. yoelii* 17XNL genomic DNA was generated from parasitised red blood cells (pRBC) of BALB/c mice after infection with cryopreserved *P. yoelii* 17XNL sporozoites. Py18S rRNA and PyCytB mtDNA were amplified by PCR from the *P. yoelii* genomic DNA and the amplified full length gene inserts (Figure 1B) were cloned into pGEM-T cloning vector (Invitrogen, Life Technologies Australia Pty Ltd.) using the TA-cloning method. These control plasmids were used as external standards. The Py18S standard curve was generated from a 10-fold dilution series from 10^7^ to 10^2^ plasmid copies and then a 2-fold dilution to 0.63 plasmid copies. The PyCytB standard curve was derived from a serial 10-fold dilution ranging from 10^7^ to 0.5 plasmid copies of CytB cDNA.

### 5. Primers and Probes

cDNA derived from *P. yoelii* 18S rRNA (Py18S) was quantified by RT-PCR using a custom dual-labelled probe and custom primers (Geneworks Pty. Ltd, SA). Both the probe and the primer sequences have been previously published by us ( [11], Table 1), but the quencher used on the Py18S probe was updated from TAMRA to BHQ-1. Amplification with these primers generates a 98bp fragment (Figure 1C) that contains 69 mismatches (41.5% homology) with the homologous mouse 18S rRNA sequence (Rn18S; NCBI Gene ID: 19791; Figure 2).

**Table 1:** Primer and probe sequences.

ID	Sequence
Py18S rRNA probe*	5’ 6-FAM-CTGGCCCTTTGAGAGCCCACTGATT-BHQ-1 3’
Py685F*	5’ CTTGGCTCCGCCTCGATAT 3’
Py782R*	5’ TCAAAGTAACGAGAGCCCAATG 3’
PyCytB mRNA probe	5’ 6-FAM-TGCACGCTACTGGTGCATCA-BHQ-1 3’
PyCytB Fw	5’ GGAGTGGATGGTGTTTTAGA 3’
PyCytB Rv	5’ CACCCCAATAACTCATTTGT 3’

* Sequences from [11]. The quencher on the Py18S rRNA probe dual labelling was updated from TAMRA to BHQ-1.

**Figure 2:**
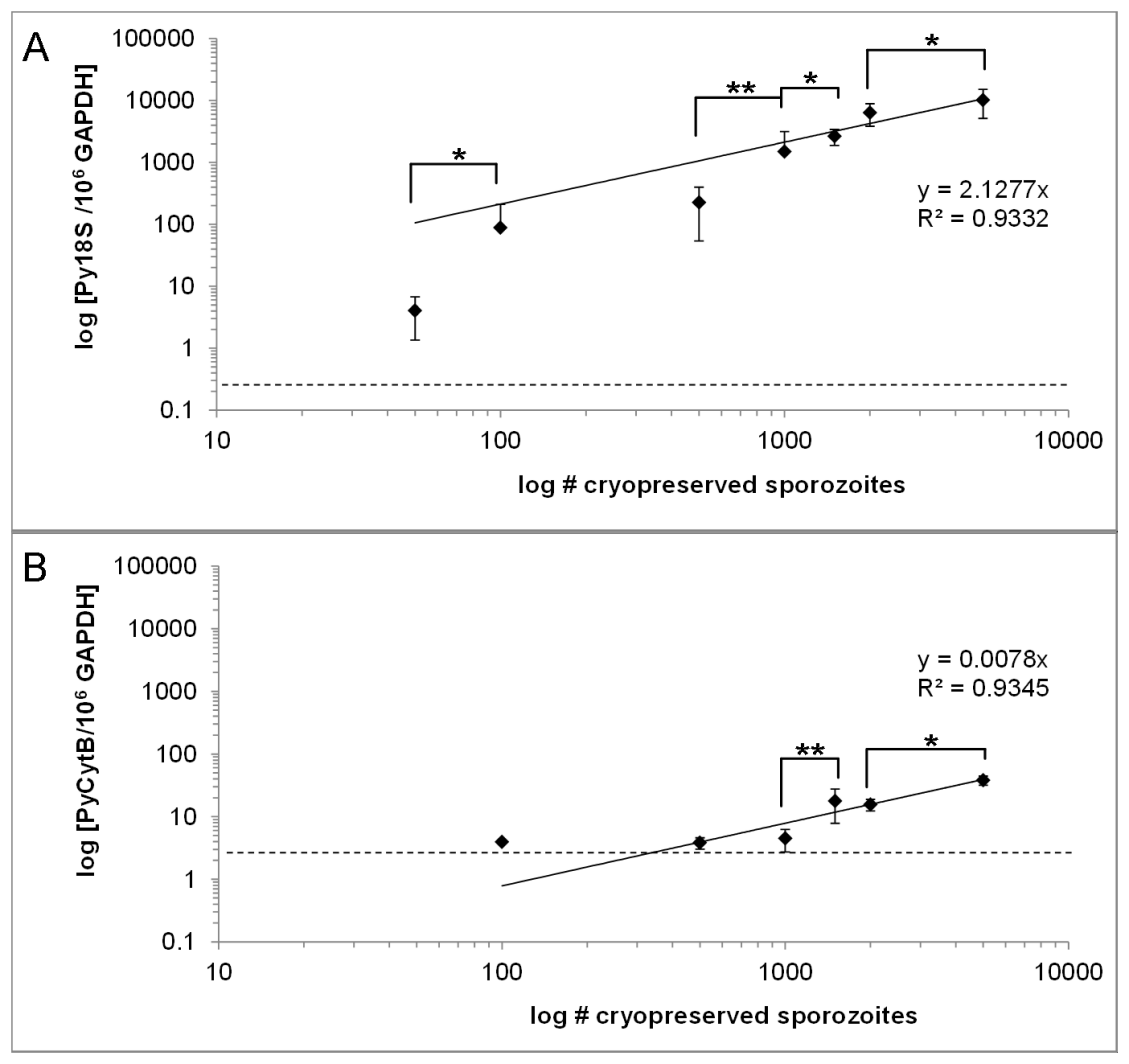
Limit of Detection after infection with different numbers of cryopreserved sporozoites. Detection of (A) Py18S rRNA or (B) PyCytB mRNA in liver RNA extracts of BALB/c mice 40h after infection with 50, 100, 500, 1000, 1500, 2000 or 5000 cryopreserved sporozoites. Data are represented as mean ‘plasmid equivalent’ for each group (n=3-7 mice/group). Two technical replicates of each mouse sample were run twice in independent qRT-PCR experiments and the mean ‘plasmid equivalent’ measured for each mouse/group was calculated. The error bars represent the mean variation between mice receiving the same sporozoite dose, calculated as the standard error for each group. A linear trendline was fitted to the data points to represent the correlation of measured values to the number of injected sporozoites. The dotted line represents the limit of detection defined as the mean value of target cDNA per 10^6^ copies GAPDH within the group of samples for which a maximum of 5% of reactions failed. Statistical significance between groups of mice receiving different sporozoite doses was evaluated using One-way ANOVA followed by two-tailed Mann-Whitney test, * p<0.05, ** p<0.005.

*P. yoelii* cytochrome B mtDNA (PyCytB) specific primers and probe were based on the previously published *P. yoelii* (17XNL) CytB sequence (PY00774; NCBI Gene ID: 3792183) and designed using the Primer3 software (http://simgene.com/Primer3). Amplification with these primers generates a 207bp fragment (Figure 1C) that contains 143 mismatches (41.6% homology) with the homologous mouse cytochrome B mtDNA sequence (Cyb561; Figure 2).

A commercially available qRT-PCR kit (TaqMan® Gene Expression Assays from Applied Biosystems, Life Technologies Australia Pty Ltd., Mulgrave, VIC) was used to amplify mouse glyceraldehyde 3-phosphate dehydrogenase (GAPDH) as a housekeeping gene for the normalisation of Py18S or PyCytB values.

### 6. qRT-PCR

Py18S and PyCytB qRT-PCR conditions were determined empirically by testing a range of primer concentrations (range 0.2µM-1µM) and probe concentrations (range 0.1µM-0.5µM) with different PCR reaction mixes in 15µl reactions with 2µl cDNA. Specifically, we evaluated different DNA polymerases (Platinum® Taq DNA Polymerase (Invitrogen, Life Technologies Australia), AmpliTaq® Fast DNA Polymerase (Applied Biosystems, Life Technologies Australia) and *taq* DNA Polymerase (GE Healthcare Life Sciences)), different reverse transcriptases (M-MLV Reverse Transcriptase (GE Healthcare), SuperScript(R) III and SuperScript® Vilo reverse transcriptase (Life Technologies Australia)) and different PCR reaction mixes (Platinum® Taq PCR Mix (Invitrogen, Life Technologies Australia), RT-PCR Master Mix (GE Healthcare Life Sciences) and TaqMan(R) Fast Advanced Master Mix (Applied Biosystems, Life Technologies Australia)). Optimal amplification was achieved with primer concentrations at 1µM, probe concentration at 250nM and Fast Advanced Master Mix using a Rotorgene 3000A PCR machine (Corbett Research, Mortlake, NSW) with the following conditions: 2 minutes at 50°C for calibration of fluorescence gain values, then denaturing at for 2 minutes at 95°C, followed by 50 cycles of 5 seconds at 95°C and 30 seconds at 60°C (data not shown).

Non-template controls (H_2_O with 10µg/ml tRNA as carrier) and liver samples of uninfected mice were included in each run.

### 7. Analysis and Statistical Evaluation

All data was acquired on the Rotorgene 3000A and analysed using Rotor-Gene software version 6.0 (both Corbett Research). The cycle at which a defined threshold of fluorescence intensity is reached (quantification cycle, C_q_) in each sample is proportional to the amount of target sequence in the extracted sample [18]. For our analysis, the threshold was set at the point of inflexion of the PCR curve. The ‘plasmid equivalents’ of target cDNA were calculated using a standard curve derived from a dilution series of control plasmid. Data generated in a qRT-PCR run was accepted only if non-template controls and samples from uninfected control mice were negative.

Since the starting amount of RNA used to generate cDNA samples was constant between all samples, qRT-PCR directly quantified the number of target molecules amplified from the extracted sample. Reference genes were used to ensure comparability between different samples, and control the quality and yield of RNA extraction and the efficiency of cDNA synthesis. The parasite liver burden in samples derived from *P. yoelii* infected or uninfected mice was quantified by calculating the ratio of ‘plasmid equivalents’ of Py18S rRNA or PyCytB mtDNA to 10^6^ units of GAPDH. The mean values and standard deviation between technical replicates and the variance between runs were calculated in Microsoft Office Excel 2007.

The limit of detection of *P. yoelii* parasites was defined as the lowest number of ‘plasmid equivalents’ which could be detected with 95% certainty for the target cDNA within liver samples of sporozoite infected mice. High accuracy of quantification of parasite cDNA in infected mouse liver samples was defined below a cut-off value of 8x10^-3^ for the ratio between standard deviation and mean of technical replicates.

## Results

### 1. RNA integrity, cDNA synthesis

To determine the integrity of extracted RNA, we assessed the ratio between the small (18S) and the large (28S) rRNA subunit by gel electrophoresis; a 28S/18S ratio between 1 and 2 is indicative of an intact RNA sample [19]. Using our RNA extraction method, there was no difference in the ratio of 28S rRNA to 18S rRNA between freshly isolated RNA samples, RNA samples frozen (-80°C) for one month and thawed immediately prior to assay, or RNA extracted from frozen (-80°C) and thawed liver homogenate (28S/18S= 1.3-1.6; Figure 1A).

Next, we generated cDNA from a pooled liver RNA sample using either SuperScript III reverse transcriptase or SuperScript® VILO^TM^ cDNA synthesis kit to optimise cDNA synthesis. The SuperScript® Vilo cDNA synthesis kit has been specifically designed to increase cDNA yields and improve the dynamic range of qRT-PCR assays by promoting the cDNA synthesis from rare RNA species in a complex sample. A 10-fold dilution series of both cDNA samples was then analysed by qRT-PCR for Py18S and PyCytB. cDNA synthesis conducted with SuperScript® VILO^TM^ cDNA synthesis kit provided 10-fold higher sensitivity (data not shown) and was thus chosen for all following cDNA synthesis reactions.

### 2. qRT-PCR optimisation

Py18S and PyCytB qRT-PCR conditions were determined empirically by testing a range of primer and probe concentrations with different DNA polymerases and their respective commercially available PCR reaction mixes (data not shown), in 15µl reactions with 2µl cDNA. Optimal amplification was achieved with primer concentrations at 1µM, probe concentration at 250nM and Fast Advanced Master Mix (data not shown) using a Rotorgene 3000 PCR machine (Corbett Research, Mortlake, NSW).

### 3. Assay Validation

To validate the quantitative RT-PCR (qRT-PCR) assay, we assessed the primer efficiency, dynamic range, and specificity of the reaction using plasmid constructs containing Py18S or PyCytB cDNA, or a pooled sample of cDNA derived from liver homogenates of mice infected with varying amounts of sporozoites. Sensitivity and accuracy was assessed from liver RNA samples of mice infected with a titration of cryopreserved sporozoites.

#### 1. Efficiency of PCR amplification

The efficiency of amplification with Py18S or PyCytB primer sets under the optimised qRT-PCR conditions was determined using a 2-fold dilution series of a pool of cDNA samples derived from mouse liver RNA extracts with varying parasite burden. Optimally, a doubling in the amount of cDNA in each amplification cycle results in exponential amplification to the base of 2. Thus, the efficiency of the amplification can be calculated by determining the slope of the curve described by the C_q_ values on the y-axis and the log of the dilution factor on the x-axis and completing following equation: amplification efficiency = 10^(-(1/slope)) [18]. Optimally, the value for efficiency is 2 (+/-10%). Values between 1 and 2 indicate suboptimal amplification and values higher than 2.2 are generally the result of primer dimers or non-specific amplicons. The mean C_q_ values of two independent PCR runs with two technical replicates and the resulting primer efficiency for Py18S, PyCytB and GAPDH are presented in Table 1. Both primer pairs showed optimal efficiency (Py18S: 2.04; PyCytB. 2.02) (Table 1). Furthermore, the difference in C_q_ values between target genes and the reference gene (GAPDH) is consistent across all sample dilutions, demonstrating the suitability of normalisation of our target genes with GAPDH (18S/GAPDH: 2.22±0.15; CytB/GAPDH: 2.29±0.17; 95% confidence interval) (Table 1).

#### 2. Dynamic Range

The dynamic range and analytical sensitivity of the qRT-PCR assay for Py18S was determined using a dilution series of control plasmid encoding Py18S covering seven log_10_ concentrations. The derived standard curve for Py18S was linear (R^2^=0.998) over the range of 1.25 to 10^7^ copies of template Py18S cDNA (Figure 1A). At 0.63 copies the qPCR was negative, indicating (analytical) sensitivity down to 1.25 copies. The mean C_q_ value at the lowest concentration was 39.03±0.23 (95% confidence interval) and the variation at this concentration is within the range of variation calculated for the whole linear interval (0.18-0.30, 95% confidence interval).

Similarly, the standard curve for our PyCytB qRT-PCR assay derived from a serial dilution series of PyCytB control plasmid over a range of 1 to 10^7^ copies of template PyCytB cDNA was linear (R^2^=0.9965) down to 1 copy (Figure 1B). The mean C_q_ value at the limit of analytical sensitivity was 41.72±0.55 and the variation across the whole linear interval ranged from 0.08 to 1.27 (95% confidence interval).

These Py18S and PyCytB standard curves were determined using duplicate samples for each plasmid copy number and repeated over three consecutive days to assess technical variability of the qRT-PCR assay. The repeatability is expressed as the standard deviation (s) between technical replicates within one run, the mean standard deviation for all three runs is displayed by the error bars in Figure 1. High reproducibility was demonstrated by a low mean variance (s^2^) between runs of the qRT-PCR assay for Py18S and for PyCytB (Py18S: 0.07±0.02; PyCytB: 0.52±0.49; 95% confidence interval).

#### 3. Capacity to detect *P*. *yoelii* liver stage parasites

The capacity of the assay to quantitate a wide range of liver stage parasite burdens was determined in BALB/c mice (3-7 per group) injected with 50, 100, 500, 1000, 1500, 2000 and 5000 cryopreserved *P. yoelii* sporozoites. We previously established that 50 cryopreserved sporozoites are sufficient to infect 100% of BALB/c mice and that the pre-patent period in all mouse cohorts was 6 days (data not shown). Standard curves for Py18S and PyCytB covering plasmid copy numbers from 1 to 10^6^ were used to determine the precise amount of parasite-derived cDNA molecules in mouse liver samples by linear regression analysis. In two independent runs Py18S qRT-PCR detected parasite-derived cDNA in 95% of replicates from mice infected with 50 sporozoites. The highest C_q_ value detectable with at least 95% certainty was 38.5, corresponding to 1.5 Py18S molecules per reaction or 37.5 ‘plasmid equivalents’ per 2.5µg extracted RNA; thus these values represent the limit of detection for our 18S qRT-PCR method (Figure 2). In all other *P. yoelii* infected mice the detected parasite burdens were well above the limit of detection. The amount of Py18S detected in mice infected with 5000 cryopreserved sporozoites is significantly different from mice infected with 2000 cryopreserved sporozoites (*p*=0.0286), and likewise for the amount measured in mice injected with 1500 versus 1000 cryopreserved sporozoites (*p*=0.0159), 1000 versus 500 cryopreserved sporozoites (*p*=0.0079) and 100 versus 50 cryopreserved sporozoites (*p*=0.0357, Figure 2A). Linear regression analysis shows that the number of injected sporozoites is correlated to the number of detected parasite-derived cDNA (R^2^=0.93; Figure 2A). Although the range of detected Py1S was larger in groups receiving low dose sporozoite inocula (50-500 cryopreserved sporozoites), there was no inverse correlation between infective dose and variation between different mice of the same group. Thus, our Py18S qRT-PCR assay can detect infection from as few as 50 cryopreserved sporozoites and reliably distinguish infected from uninfected/protected animals after challenge with 100 cryopreserved sporozoites, as well as distinguish between parasite burdens after low (50-500 sporozoite) or high (1000-5000 sporozoites) dose infection.

PyCytB qRT-PCR could not detect parasite-derived cDNA in liver samples of mice injected with 50 cryopreserved sporozoites and also failed in 83% of replicates from mice injected with 100 sporozoites, 40% of replicates from mice injected with 500 sporozoites and 28.6% of replicates from mice injected with 1000 replicates (Figure 2B). The highest C_q_ value detectable with at least 95% certainty was 37.8, corresponding to 10.3 molecules of PyCytB per reaction or 257.5 ‘plasmid equivalents’ per 2.5µg extracted RNA. Thus, infection with 100 sporozoites is detectable, but infection with at least 1500 sporozoites is necessary to reliably distinguish infected from uninfected/protected mice using PyCytB as a target gene in our qRT-PCR assay. The amount of PyCytB detected in mice infected with 5000 cryopreserved sporozoites is significantly different from mice infected with 2000 cryopreserved sporozoites (*p*=0.0195), as is the amount measured after infection with 1500 versus 1000 cryopreserved sporozoites (*p*=0.0061; Figure 2B). However, parasite burden after infection with a range of low doses of less than 500 cryopreserved infectious sporozoites was not significantly distinguishable. Although, linear regression analysis for PyCytB qRT-PCR determines a strong correlation between the level of sporozoite inoculum and detection of parasite-derived cDNA (R^2^=0.93; Figure 2B), clinical sensitivity for PyCytB is considerably lower than for Py18S (56.7% compared to 97%).

**Figure 3:**
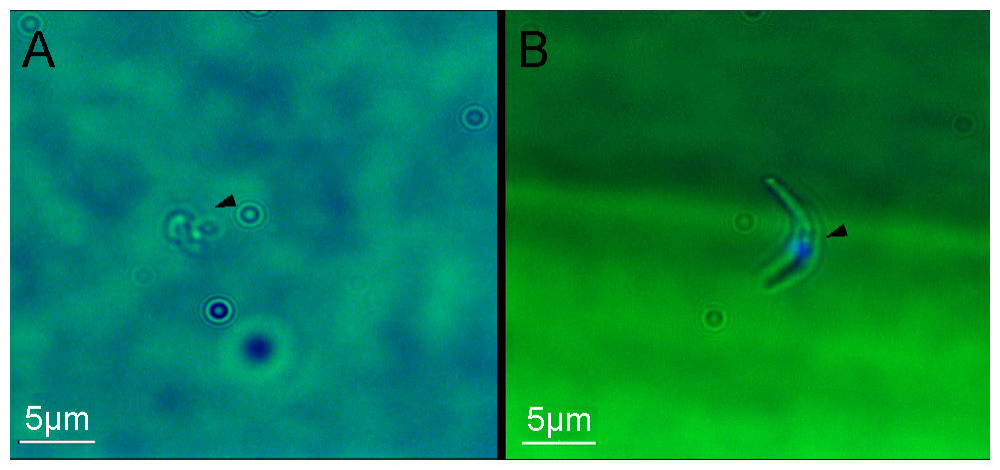
Detection of infectious versus inactivated sporozoites. Viability of cryopreserved sporozoites (**A**) and inactivation of heat-treated sporozoites (**B**) was confirmed by fluorescence microscopy (DeltaVision Core Microscope System with Coolsnap HQ camera and 100x/1.40 oil objective) using Sytox Blue (0.1% in PBS).

To confirm that all parasites detected by Py18S qRT-PCR are derived from infectious sporozoites, which have invaded and multiplied within hepatocytes, we injected mice with 2500 cryopreserved/live infectious or heat-inactivated sporozoites. Fluorescence microscopy confirmed that the majority of sporozoites from the cryopreserved infectious inoculum were live (by mobility and ability to exclude Sytox Blue) (Figure 3A), and all sporozoites found in the heat-inactivated sample were dead (by loss of mobility and Sytox Blue staining of DNA) (Figure 3B). Parasite rRNA was below the limit of detection in mice inoculated with heat-inactivated sporozoites, confirming that only viable sporozoites which have invaded and multiplied in the liver are detectable.

Notably, GAPDH qRT-PCR curves did not exhibit changes in expression between samples from a wide range of experimental conditions, indicating that amplification rates were not influenced by the varying amount of parasite cDNA present in different samples (Figure 3).

#### 4. Specificity and Accuracy

**Figure 4:**
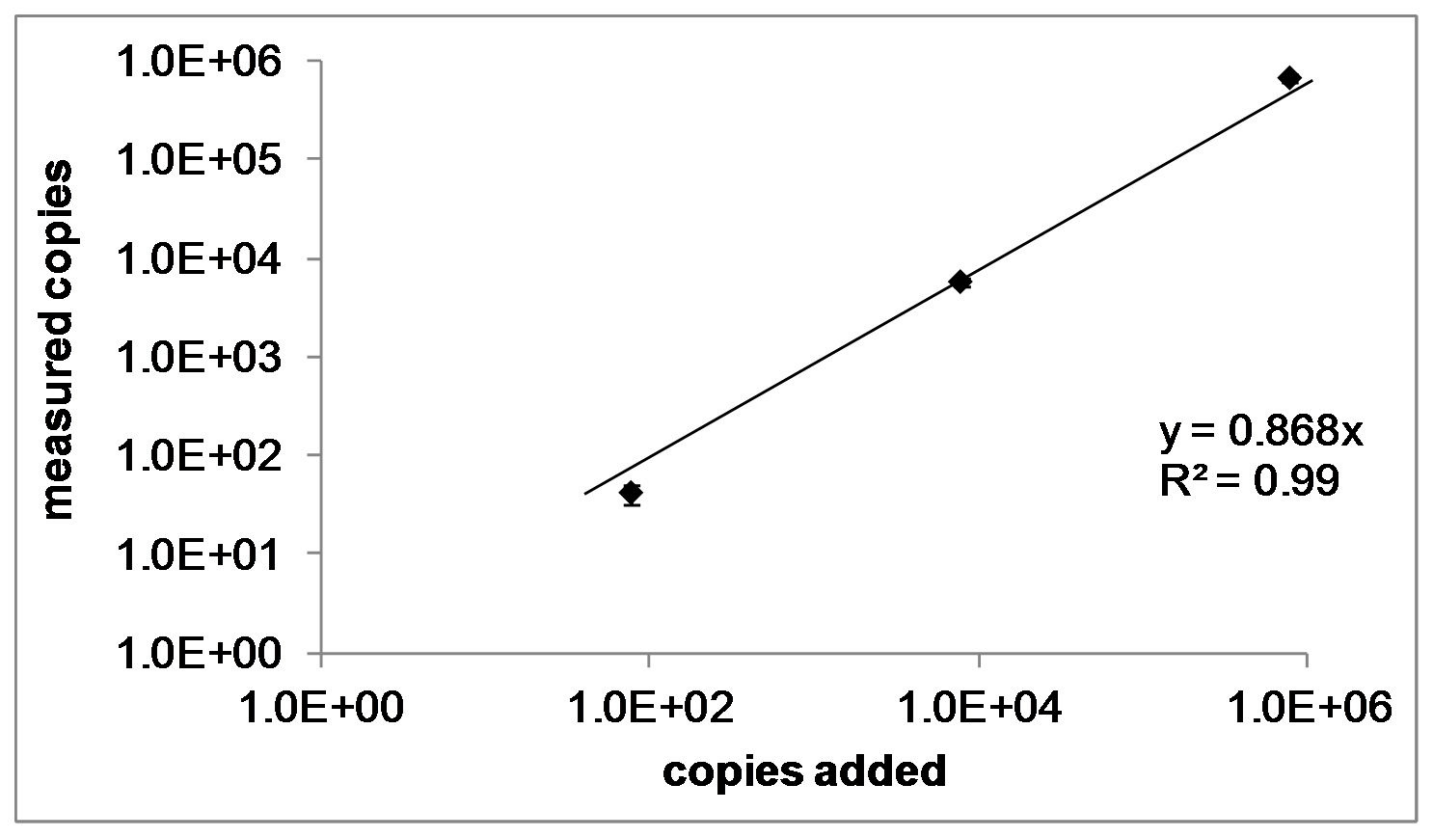
Recovery rate. A cDNA sample extracted from the liver of a naïve mouse was spiked with 7x10^5^, 7x10^3^ or 70 ‘plasmid equivalents’ of Py18S control plasmid. C_q_ values of spiked samples were plotted on the y-axis against the given copy number of plasmid. Linear regression analysis reveals a robust linear correlation of calculated to given copy numbers (R^2^=0.99). Data are presented as mean values of three technical replicates; error bars represent the standard deviation.

To confirm that only parasite rRNA was amplified and to determine the effects of abundant mouse rRNA on the qRT-PCR reaction, we spiked a cDNA sample from a naïve BALB/c mouse with specific copy numbers of Py18S control plasmid and measured the resulting C_q_ values. The recovery rate was between 69-98%, with higher accuracy in higher copy number ranges. Linear regression analysis confirmed a robust linear correlation of measured to known DNA amounts (R^2^=0.99) (Figure 4). Py18S was not amplified from uninfected mouse samples, demonstrating 100% diagnostic specificity, thus indicating that the qRT-PCR specifically amplifies 18S cDNA rather than non-specific targets.

**Figure 5:**
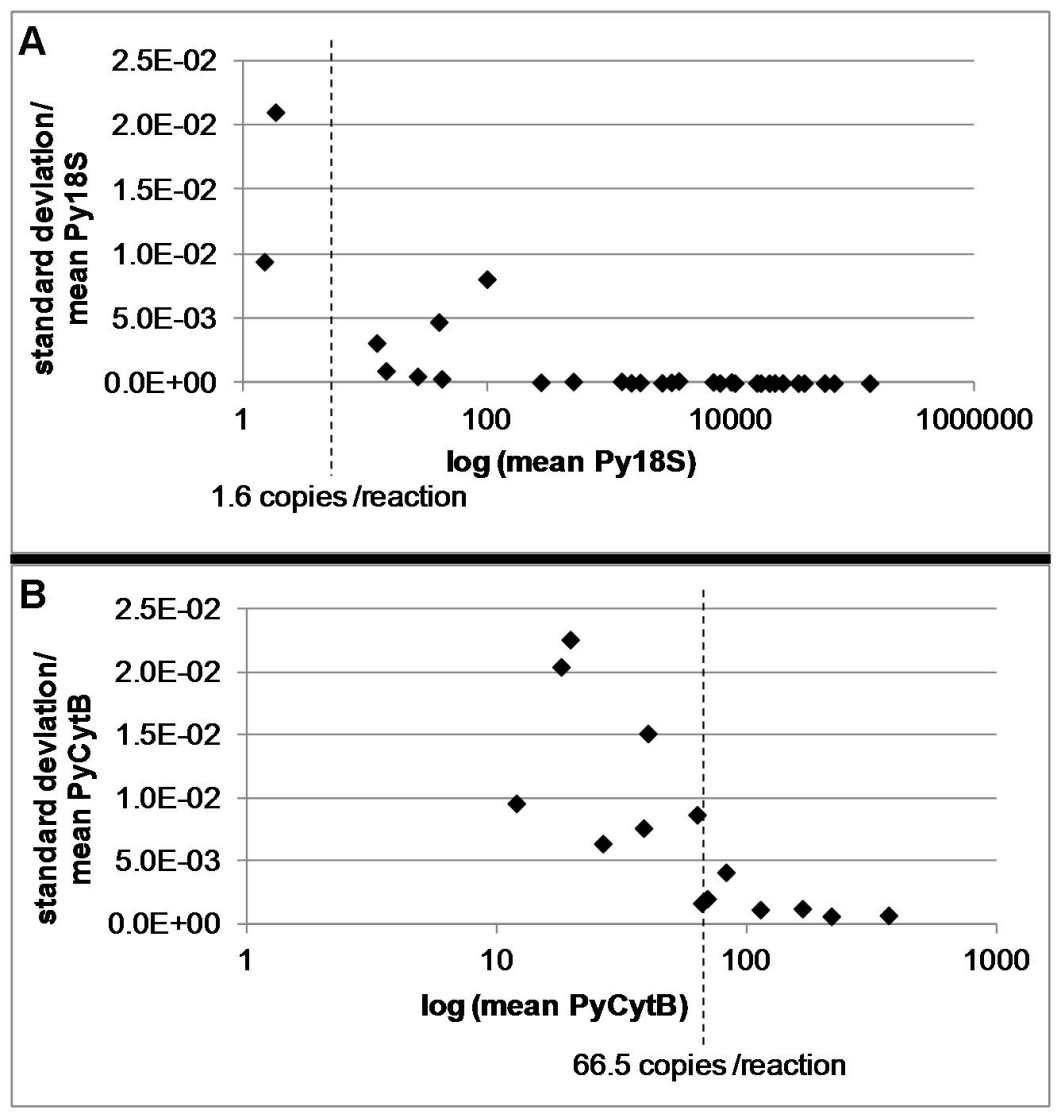
Accuracy of parasite cDNA detection. The standard deviation for four replicates of one sample was divided by the mean value for the target gene and plotted against the log of the mean parasite-derived cDNA copies/reaction. Each point represents the mean of four replicates of one mouse liver cDNA sample. A cut-off value of 8x10^-3^ for the ratio between standard deviation and mean is suggested for accurate quantitation. The dotted line marks the mean value between the highest amount of parasite cDNA measured with a standard deviation/mean ratio below 8x10^-3^ and the highest amount of parasite cDNA measured with a standard deviation/mean ratio above 8x10^-3^, for (A) Py18S qRT-PCR (1.6 copies/reaction) and for (B) PyCytB qRT-PCR (66.5 copies/reaction).

The accuracy of our qRT-PCR assay for both Py18S and PyCytB target genes was determined by calculating the ratio of standard deviation to mean copy number for four replicates from two independent PCR runs and plotting it against the log_10_ of mean parasite-derived cDNA molecules as described in [20] (Figure 5). Although low levels of parasite cDNA could be detected in both assays, accuracy decreases with decreasing parasite numbers as evidenced by the increase in standard deviation between replicates. To ensure high accuracy of parasite quantitation we suggest a cut-off at a standard deviation/mean value of 8x10^-3^, resulting in a limit for high accuracy at 1.6 molecules/reaction for our Py18S qRT-PCR assay and at 66.5 molecules/reaction for our PyCytB qRT-PCR assay.

## Discussion

Rodent malaria parasites are widely used as model systems for the study of the biology and pathology of *Plasmodium* *spp.*, as well as for the evaluation of candidate vaccines or drugs. *P. yoelii* is a robust model for pre-erythrocytic stage development and ensuing protective immunity, and is often used in studies evaluating vaccine or drug candidates [21,22].

Microscopic methods have been used to detect *Plasmodium* *spp.* liver stages and describe the morphological development of early and late hepatic stages [7], but are time-consuming, insensitive, not accurately quantifiable, and not compatible with high-throughput approaches. Recently, qRT-PCR and bioluminescence or fluorescence measurements using transgenic parasites have shown promising results in evaluating liver stage parasite burden with high sensitivity [1,3,10]. Fluorescent parasites have been generated and used for the study of *P. yoelii* and *P. berghei* liver stages in different mouse strains [8]. In one study, liver parasite burden after infection with GFP transgene parasites was assessed by fluorescence microscopy of perfused liver sections, revealing that inoculation with 3x10^6^ sporozoites resulted in 1000 liver stage s/cm^2^. In other studies, GFP transgene parasites were used to isolate infected hepatocytes by FACS sorting and to detect parasite burden by intravital imaging through the peritoneal cavity [8,23]. While these results demonstrate the value of GFP transgenic parasites for cell culture and biological characterisation of liver-stage parasites and infected hepatocytes, issues with promoter choice and variance in GFP expression [24] have limited the use of GFP parasites for the evaluation of vaccine or drug induced reduction of parasite burden *in vivo*. Luciferase expressing parasites on the other hand have been used to evaluate the protective capacity of low dose whole-parasite infection [25], the effect of blood-stage infection on protective immunity against parasite liver-stages [1] and the effect of various drugs [1] using *in vivo* imaging. However, the luminescence signal associated with infection of less than 1000 sporozoites was found to be indistinguishable from background luminescence [1]. Moreover, the sensitivity of bioluminescence imaging is limited by the low level expression of luciferase in transgene parasites, so negative luminescence signals cannot be definitively associated with low parasite numbers in the liver or claims of sterile protection [25].

PCR-based methods have been described for the detection and quantification of rodent *Plasmodium* spp. liver stages with different RNA targets, including 28S rRNA and 18S rRNA [10,11,13]. For example, one study in 1996 reported the detection of liver-stage parasite burden after infection with only 200 sporozoites, but the results show that with these inoculation numbers only 50% of infected mice were positive for parasite rRNA in their qPCR assays, although all developed blood-stage parasitaemia following challenge with 200 sporozoites [13]. In another study, parasite derived cDNA was detected in mice infected with 25 freshly isolated sporozoites or the bite of one infected mosquito [26].

Despite these technological developments, failure to reliably detect low level liver-stage parasite infection directly impacts on the standard infective dose used for challenge studies, which can be well over 5,000 sporozoites [11]. The use of high inoculums may overwhelm the host immune system resulting in a failure to detect any partial protective effect at the liver stage and thus provide incorrect and misleading results for partially effective vaccines or drug candidates. Furthermore, mosquito parasite loads can vary greatly [26] and neither infectivity nor transmission of sporozoites through mosquito bites can be quantified making the accurate assessment of the efficacy of candidate drug or vaccines problematic. Improved techniques for the production, dissection, purification and cryopreservation of infectious *Plasmodium* *spp.* sporozoites from mosquito salivary glands offer the possibility of highly accurate and repeatable challenge studies [27]. Indeed, associated studies have demonstrated that inoculation of sporozoites by mosquito bite is much less efficient than inoculation by syringe [26] and sporozoite infection via the intravenous route is generally found to be the most successful means of achieving high hepatocyte infection rates [27,28]. However, cryopreservation decreases the infectivity of sporozoites and 7.5 times more cryopreserved than fresh sporozoites are required to infect 50% of mice [29].

Using our qRT-PCR assay we were able to detect sporozoites in 100% of mice infected with as few as 50 cryopreserved sporozoites (corresponding to approximately 7 freshly isolated sporozoites [29]) and reliably distinguish infected from uninfected/protected mice after challenge with 100 cryopreserved sporozoites, as well as distinguish between parasite loads derived from low or high dose inocula. This assay therefore allows for a reduction of the standard infective dose for challenge studies to 100 cryopreserved sporozoites or 10-15 freshly isolated sporozoites. Since mosquitoes are thought to transmit around 100 infectious sporozoites per bite, and challenge by intravenous injection of the bite of at least 2 and typically 5 mosquitoes is required for 100% infectivity, our assay permits for the first time accurate quantitation of liver stage parasites following low and biologically relevant parasite challenge. We also achieved high accuracy for the quantitation of very low amounts of parasite cDNA (1.6 copies of Py18S/100ng extracted RNA) in liver samples of infected mice.

Previously qRT-PCR methods were not published with high detail or consistency in terminology, making it difficult to compare between the different methods [10,11,30,31]. The MIQE guidelines were introduced in 2009 in order to target the reliability of results to help ensure the integrity of the scientific literature, promote consistency between laboratories, and increase experimental transparency by describing the minimum information necessary and provide authors, reviewers and editors with these specifications [14,32]. In conformance with these guidelines, we validated our optimised qRT-PCR assay by determining the primer efficiency, dynamic range, specificity and accuracy of parasite cDNA detection. We report a high dynamic range (covering seven log_10_ concentrations) and high recovery rates (69-98%) of parasite 18S rRNA within the abundance of mouse RNA, by using SuperScript® Vilo for cDNA synthesis and Fast Advanced Mix for qRT-PCR reaction. The use of these improved conditions promotes detection of rare RNA species and resulted in 10-fold increased sensitivity and dynamic range of the qRT-PCR, in our hands.

Other gene targets have been suggested to confer higher sensitivity due to higher abundance within the parasite genome offering lower limits of detectable parasite numbers. With copy numbers ranging from 30 to 100 per parasite in *Plasmodium* spp., mtDNA was recently proposed as superior target to 18S rRNA [17]. The cytochrome B (CytB) gene is one of the three protein-encoding genes found in mtDNA, and has been widely used to study phylogenetics and evolutionary relationships among plasmodia [33]. For example, in one study, CytB qRT-PCR was shown to be 16% more sensitive than 18S qRT-PCR in human blood samples and could be used to detect and distinguish various *Plasmodium* species in human saliva and urine samples [17,34]. However, in our qRT-PCR assay, detection of CytB in infected mouse liver samples was much less sensitive than detection of Py18S rRNA with 15 times higher sporozoite numbers being required to ensure reliable distinction of infected and uninfected/protected individuals and 40 times higher numbers of parasite cDNA molecules required for accurate quantitation.

In conclusion, we have established 18S rRNA as a reliable target for a highly sensitive qRT-PCR assay which allows the detection and accurate quantitation of *P. yoelii* liver stage parasite burden after inoculation with as few as 50 sporozoites, a much smaller dose than previously described and in the same range as dose following natural challenge in the field situation. This assay presents a valuable tool for the evaluation of candidate drug and vaccines targeting pre-erythrocytic parasite stages in the rodent model and allows the use of parasite numbers close to those naturally occurring in field challenge.

## Supporting Information

Figure S1 **RNA integrity, target genes and amplicons.** (A) The integrity of RNA either, freshly extracted (lane 2), stored for one month after extraction at -80°C (lane 3), or freshly extracted from a liver homogenate stored at -80°C for one month (lane 4) was assessed using gel electrophoresis on a 0.8% agarose gel. The densities of the large and small subunits of rRNA were determined using Quantity One 1-D analysis software (Bio-Rad Laboratories Pty. Ltd., NSW), and the ratio was calculated. The 1kb DNA ladder from New England Biolabs was used as a size reference marker (lanes 1 and 5). (B) Py18S rRNA (lane 2) and PyCytB mtDNA (lane 3) were amplified from *Plasmodium yoelii* genomic DNA and analysed by gel-electrophoresis on a 0.8% agarose gel. (C) Py18S rRNA (lane 2) and CytB mtDNA (lane 3) amplicons after 50 cycles of qRT-PCR. The 1kb DNA ladder from New England Biolabs is used as a size reference (lane 1).
(TIF)

Figure S2 **Primers and probe sequence alignment.** (A) Alignment of 18S primers (reverse primer: Py782R; forward primer: Py685F) and probe with Py18S rRNA (Py18S) and mouse 18S rRNA (Rn18S). (B) Alignment of CytB primers (reverse primer: CytB Rv; forward primer: CytB Fw) and probe with PyCytB (PY00774) and mouse Cytochrome B (Cyb561). The analysis was performed using ClustalW2 (www.ebi.ac.uk).
(TIF)

Figure S3 Reference genes. The qRT-PCR curves of mouse GAPDH across all evaluated mouse liver RNA samples (N=62 data points) are presented together with the threshold (defined at the point of inflexion of the PCR curves) at which C_q_ values were determined.
(TIF)

Table S1 Primer efficiency. qRT-PCR was performed on a 2-fold dilution series of a pool (n=25) of mouse liver cDNA samples infected with ***P. yoelii***. *The slope of the curve described by the C_q_ values on the y-axis and the log of the dilution factor on the x-axis was determined using Microsoft Office Excel 2007. Primer efficiency was calculated by completing following equation: amplification efficiency= 10^ (-(1/slope)). The ratio of C_q_ values for the target cDNA and GAPDH was calculated for each dilution, in order to compare efficiencies between target and reference genes.
(DOCX)
