# Supplementary figures and images for: Highly Sensitive Quantitative Real-Time PCR for the Detection of Plasmodium Liver-Stage Parasite Burden following Low-Dose Sporozoite Challenge
Source: PLoS One. 2013 Oct 2;8(10):e77811. doi: 10.1371/journal.pone.0077811 (PMC3788780; doi:10.1371/journal.pone.0077811)

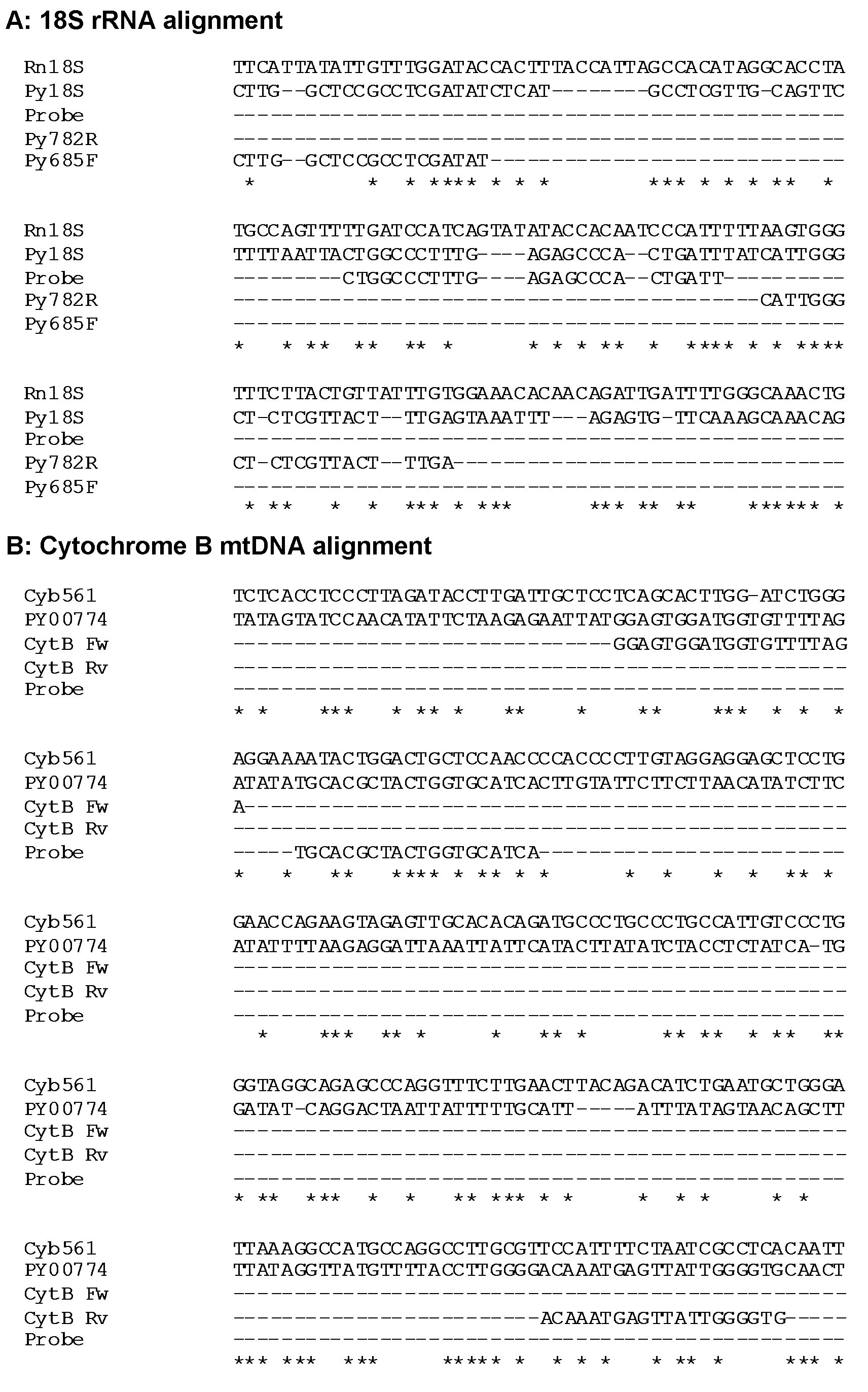

Supplement: Figure S2 — Primers and probe sequence alignment. (A) Alignment of 18S primers (reverse primer: Py782R; forward primer: Py685F) and probe with Py18S rRNA (Py18S) and mouse 18S rRNA (Rn18S). (B) Alignment of CytB primers (reverse primer: CytB Rv; forward primer: CytB Fw) and probe with PyCytB (PY00774) and mouse Cytochrome B (Cyb561). The analysis was performed using ClustalW2 (www.ebi.ac.uk). (TIF) [file pone.0077811.s002.tif]

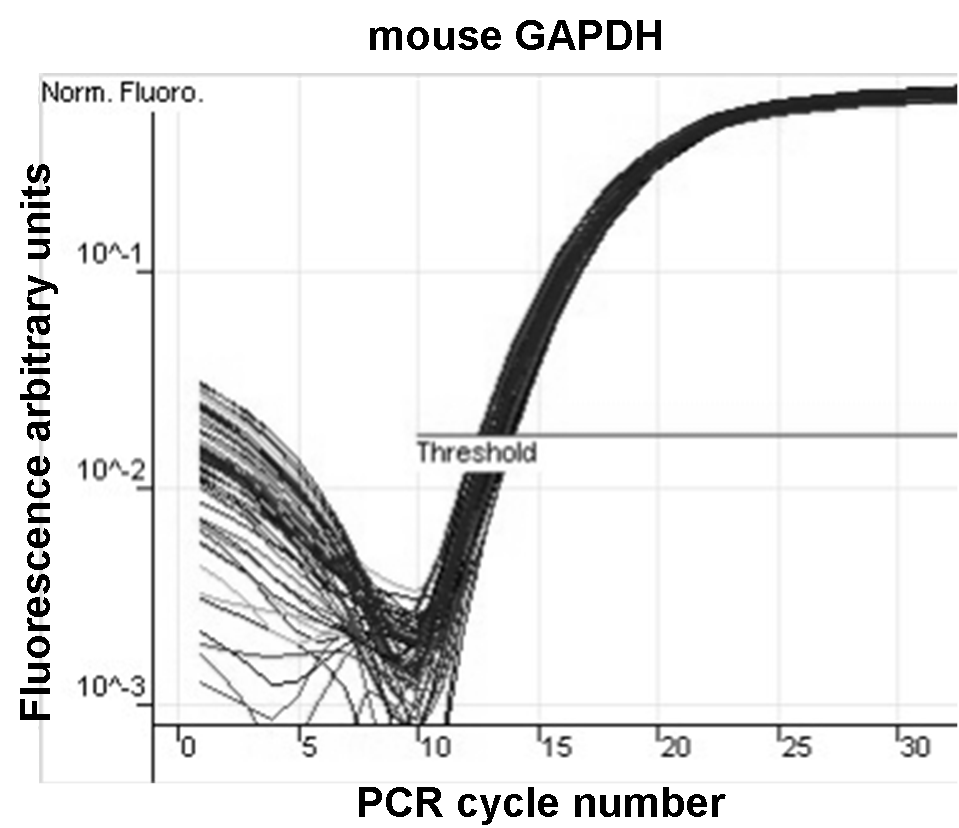

Supplement: Figure S3 — The qRT-PCR curves of mouse GAPDH across all evaluated mouse liver RNA samples (N=62 data points) are presented together with the threshold (defined at the point of inflexion of the PCR curves) at which Cq values were determined. (TIF) [file pone.0077811.s003.tif]
