# Supplementary material for: Highly Sensitive Quantitative Real-Time PCR for the Detection of Plasmodium Liver-Stage Parasite Burden following Low-Dose Sporozoite Challenge
Source: PLoS One. 2013 Oct 2;8(10):e77811. doi: 10.1371/journal.pone.0077811 (PMC3788780; doi:10.1371/journal.pone.0077811)
Supplement: Supplementary file 2 [file pone.0077811.s004.docx]

	dilution	1.00	0.50	0.25	0.13	0.06	0.03	0.02	Slope*	efficiency*
	log	0.00	-0.30	-0.60	-0.90	-1.20	-1.51	-1.81		
primer	**PyCytB**	34.25	35.68	35.05	34.79	37.90	38.45	40.64	-3.27	2.02
primer	**Py18S**	33.41	33.48	34.90	33.99	36.61	37.27	n.d.	-3.23	2.04
	**GAPDH**	n.d.	13.71	14.94	16.22	16.95	18.07	18.85	-3.56	1.91
	**18S/GAPDH**		2.44	2.34	2.10	2.16	2.06			
	**CytB/GAPDH**		2.60	2.35	2.14	2.24	2.13	2.16		
